# Validation of Ultra-Widefield Autofluorescence Imaging as an Endpoint for Disease Progression in Choroideremia: A Comparative Study

**DOI:** 10.1016/j.xops.2026.101331

**Published:** 2026-07-24

**Authors:** Mechteld C.S. van Olden, Jeroen A.A.H. Pas, Suzanne Yzer, Carel B. Hoyng, Thomas Theelen

**Affiliations:** 1 Department of Ophthalmology, Radboud University Medical Center, Nijmegen, The Netherlands; 2 Donders Institute for Brain, Cognition and Behavior, Radboud University Medical Center, Nijmegen, The Netherlands

**Keywords:** Choroideremia, Fundus autofluorescence, Ultra-widefield

## Abstract

**Objective:**

Promising gene therapy studies in patients with choroideremia (CHM) have failed to meet the primary endpoint of best-corrected visual acuity, but other therapeutic effects in the peripheral fundus may not have been discovered. Ultra-widefield fundus autofluorescence (UWF-FAF) is able to capture peripheral retinal changes as potential structural endpoint. We compared UWF-FAF with conventional FAF in patients with CHM to evaluate its potential as additional imaging modality for future clinical trials.

**Design:**

Retrospective cohort study.Subjects: Participants with genetically confirmed CHM and both central 30° and 55° FAF and UWF-FAF imaging available.

**Methods:**

Conventional FAF (30° or 55°) and UWF-FAF images obtained on the same day were retrospectively aligned for direct comparison and then analyzed. Two independent graders outlined atrophic areas and preserved retinal pigment epithelium (RPE) islands. Between-modality differences with standard errors, 95% confidence intervals (CIs), and 2-sided *P* values (α = 0.05) were calculated. Intergrader agreement was assessed using intraclass correlation coefficients (ICCs).

**Main Outcome Measures:**

Between-modality differences in area measurements of FAF and UWF-FAF with standard errors, 95% CIs and ICCs.

**Results:**

Ninety-six FAF images from 15 patients were included (35 FAF 30°, 20 FAF 55°, and 41 UWF-FAF). Intergrader ICCs ranged from 0.625 to 0.998, indicating good-to-excellent agreement. Within the 30° field, no significant difference was found for the total cohort (0.01; 95% CI, –0.02 to 0.04; *P* = 0.438) or for atrophy (–0.06; 95% CI, –0.14 to 0.01; *P* = 0.089), whereas preserved RPE islands differed significantly (0.04; 95% CI, 0.01–0.06; *P* = .005). Within the 55° field, differences did not reach statistical significance for the total cohort (0.08; 95% CI, –0.01 to 0.18; *P* = 0.077), atrophy (0.10; 95% CI, –0.09 to 0.28; *P* = 0.260), or islands (0.07; 95% CI, –0.001 to 0.15; *P* = 0.054).

**Conclusion:**

In our current study UWF-FAF provided comprehensive atrophy measurements quantitatively comparable to conventional FAF in CHM, supporting its usefulness as a structural clinical trial endpoint for disease progression. Due to significant differences in preserved RPE island measurements the modalities should not be used interchangeably.

**Financial Disclosure(s):**

The authors have no proprietary or commercial interest in any materials discussed in this article.

Inherited retinal diseases (IRDs) are genetically driven retinal disorders that slowly progress to serious degeneration of the outer retina and the retinal pigment epithelium (RPE), often resulting in significant visual function loss.^1^ Taking into consideration the upcoming gene therapies for IRDs and the need for patient counseling according to visual impairment, a comprehensive monitoring of disease progression is desired. Best-corrected visual acuity (BCVA), a widely accepted clinical endpoint for many disorders, does not seem to be a suitable progression marker in IRDs because it does not equivalently reflect the progression throughout the disease.^2^^,^^3^ Fundus autofluorescence (FAF), on the contrary, is a biomarker of remarkable importance in studying IRDs because it enables assessment of the retina–RPE interface and allows monitoring of RPE atrophy.^4^, ^5^, ^6^ However, standard FAF imaging is typically limited to the central 30° to 55° of the fundus with insufficient information on the peripheral disease-related fundus changes.^7^ In contrast, ultra-widefield (UWF) imaging provides wide-ranging visualization of fundus changes up to 200° of retinal eccentricity for both FAF and color fundus images, which significantly exceeds conventional imaging field size, hence allowing comprehensive monitoring of disease progression.^8^^,^^9^

Choroideremia (CHM) is an IRD caused by mutations in the *CHM* gene, which encodes Rab escort protein 1,^10^ clinically characterized by both central and peripheral fundus impairment. The absence or deficiency of Rab escort protein 1 leads to progressive degeneration of photoreceptors, RPE, and choroid.^11^ Patients initially experience disease symptoms such as nyctalopia in childhood, followed by peripheral visual field loss in the second decade of life, ultimately leading to legal blindness with VA of <20/200 during adulthood.^12^

Several promising gene therapy studies have failed to meet the primary endpoint of BCVA,^13^ as did previous CHM trials, which may have failed not only because of insufficient therapeutic efficacy but also because therapeutic effects may have been missed if they did affect the primary endpoint applied, BCVA. Consequently, more suitable alternatives may include imaging biomarkers such as abnormalities on color fundus images, the recently proposed endpoint of the “smooth zone,” and using the atrophic area measured on FAF.^7^^,^^13^ Evaluation of the whole fundus in CHM by UWF may therefore provide an appropriate structural endpoint for monitoring disease progression in CHM.^5^^,^^14^ Such a structural endpoint may complement functional outcomes and provide additional information on anatomic disease progression.

In terms of patient counseling, which currently remains the only available form of care, UWF may offer a better overview of disease extent and facilitate patient counseling on a more reliable basis.

Therefore, the present study compared UWF-FAF imaging with conventional FAF in CHM, which, to our knowledge, has not been previously explored. By directly comparing these modalities, this study takes a first step toward validating UWF images for potential use as an endpoint in clinical trials in CHM, using conventional FAF as the established reference standard.

## Materials and Methods

### Patient Selection and Data Collection

We considered patients who visited the outpatient clinic of the Department of Ophthalmology, Radboudumc, Nijmegen, the Netherlands, for the present retrospective imaging study. Only patients with genetically confirmed CHM and both central 30° and 55° FAF imaging by Heidelberg Spectralis (Heidelberg Engineering) and UWF FAF imaging by Optos California (Optos Inc) available in their medical records were included. Besides male patients, we included female carriers of *CHM* with retinal abnormalities. The study adhered to the Tenets of the Declaration of Helsinki (2013 revision), and approval was waived as ruled by the Institutional Ethics Committee, Committee for Human-Subject Research, Radboudumc (file number 2025-17955).

Patient demographics (age, sex [male or female], and age of onset of the symptoms) and genetic mutations underlying CHM were collected from medical records. Standard FAF images at 55° (FAF 55°) and 30° (FAF 30°) were derived from the Heidelberg Eye Explorer software (version 2.6.9, Heidelberg Engineering) (Fig S1B,C, available at www.ophthalmologyscience.org). Ultra-widefield FAF (UWF-FAF) images were extracted from the Optos software, Optomap (Fig S1A). For further analysis, all images were converted to standard PNG format and exported in the highest resolution setting.

### Image Analysis

Direct quantitative comparison between FAF images of the Heidelberg Engineering and Optos devices is not feasible because of a number of confounding factors that must be considered. Although the first applies blue excitation light at 488 nm, the Optos system used in this study only allows for green excitation light at 532 nm. These wavelength differences may have a significant impact on the visualization of retinal structures, especially in the macula, because macular pigment (MP) absorbs green light significantly less than blue light.^15^ Furthermore, in case of photoreceptor impairment, green-light FAF may show less pronounced focal autofluorescence increase compared with blue-light FAF.^16^^,^^17^ Atrophy area measurements may also be affected by different chromatic aberrations in both devices and optical deformation, especially in peripheral areas in UWF imaging, which may influence quantitative measurements.^18^, ^19^, ^20^ Because of the aforementioned confounding factors, added steps in image alignment were required.

**Figure 1:**
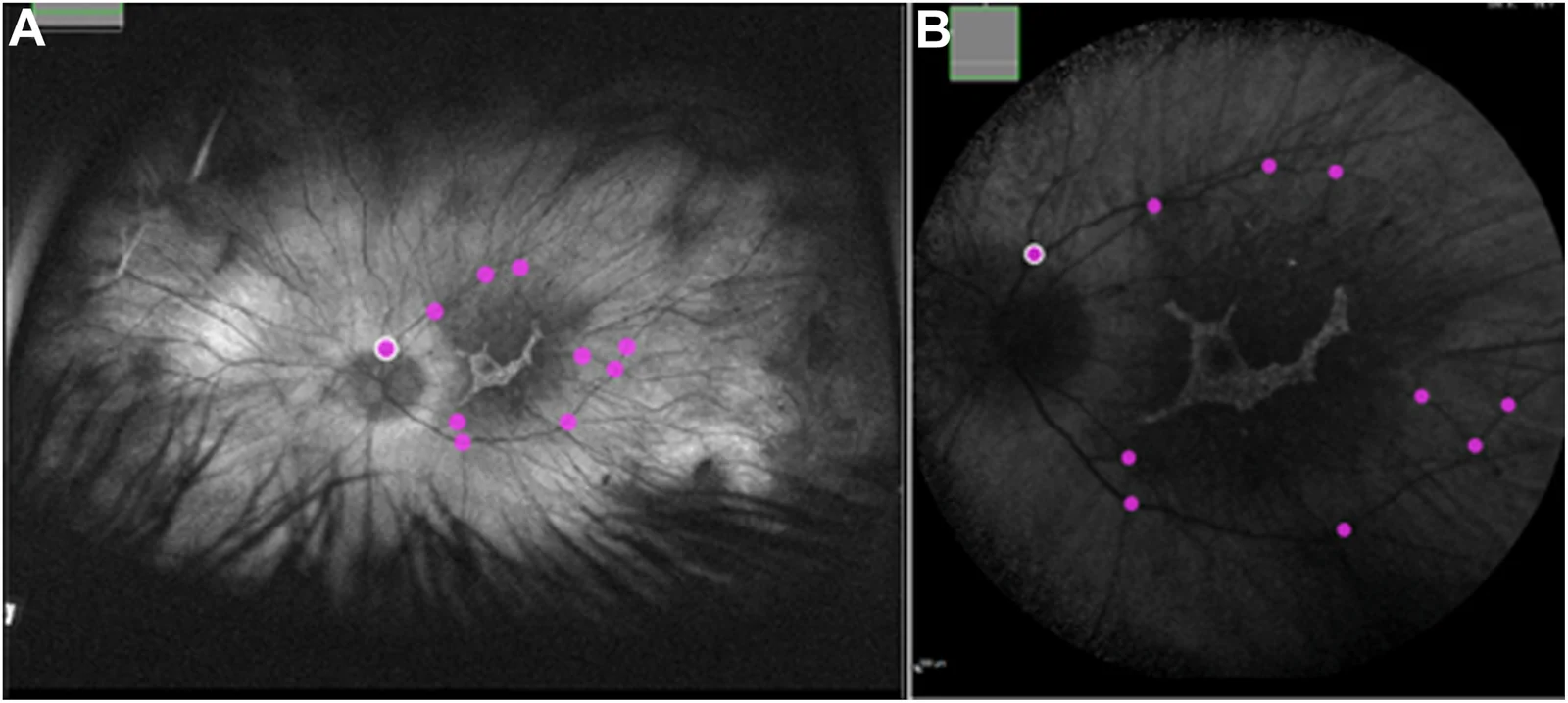
Sample of alignment between ultra-widefield (**A**) and fundus autofluorescence (**B**) images in a study patient with choroideremia. Landmark points were manually placed on corresponding retinal vessel bifurcations to warp dimensions between modalities.

ImageJ v.1.53t (National Institutes of Health) was used for image processing and analysis. For this purpose, images were exported to ImageJ as PNG before further processing. Fundus autofluorescence and UWF images for each patient were graded independently by 2 graders (M.C.S.v.O. and J.A.A.H.P.). Atrophic areas and spared islands were measured for both the left and right eyes of patients, including multiple measurements on different dates for some individuals. Atrophic area was defined as the area of RPE atrophy, whereas spared RPE islands were defined as areas of preserved autofluorescence signal within or adjacent to the atrophic region. The measurements were reported separately and combined; the latter was referred to as the total cohort. To reduce the impact of optical distortion, we have aligned images of the same patients by using a vessel-based warping plugin (*BigWarp*, v9.3.2) before analysis.^21^ To do so, we used a minimum of 5 different vessel bifurcations of retinal vessels as reference points (Fig 1).

For each patient, the FAF 30° or the FAF 55° and the UWF images obtained on the same day were aligned. Warping was conducted for both the FAF 30° with UWF and the FAF 55° with UWF, separately.

**Figure 2:**
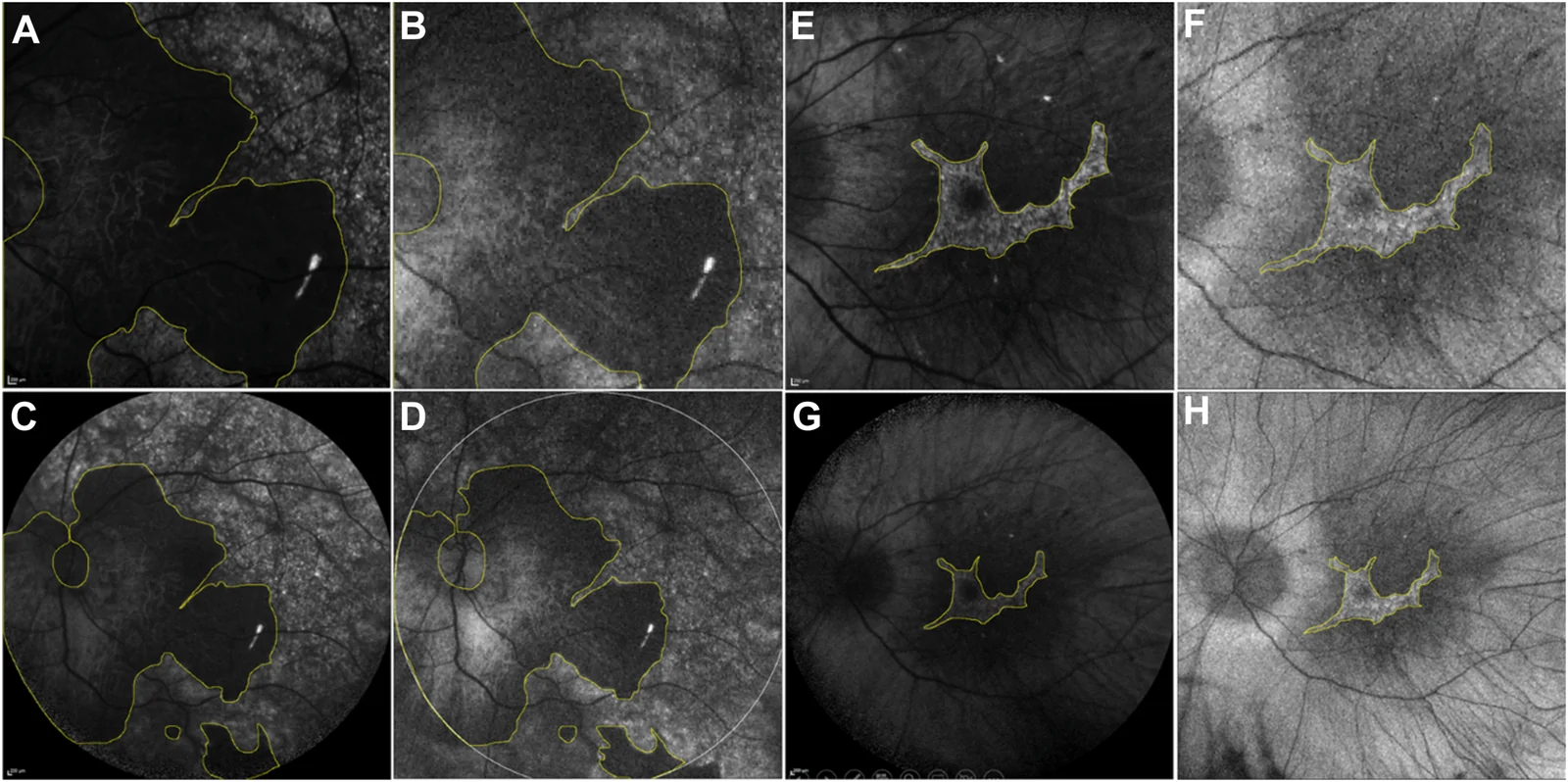
Sample of delineation of chorioretinal atrophy (**A–D**) and a spared foveal island (**E–H**) in patients with choroideremia across the different imaging modalities, as carried out in this study after image alignment by warping. The 30° fundus autofluorescence (FAF) images (**A**, **E**) and the corresponding image detail of ultra-widefield-FAF (**B**, **F**), as well as the 55° FAF images (**C**, **G**) and the equivalent image part of ultra-widefield-FAF (**D**, **H**), are depicted.

After image alignment, the retinal area visible in the FAF 30° and FAF 55° images was determined and transferred onto the warped UWF image to identify corresponding retinal regions in all imaging modalities. This allowed for comparison of atrophic areas and, thus, the same retinal area between modalities, up to the border that exceeded the visual field of the non-UWF-FAF imaging (Fig 2D). We warped each of the 2 FAF modalities with the corresponding UWF image, referred to as UWF-30 and UWF-55 hereafter.

For atrophy measurements, we used the free-form selection tool of ImageJ to trace the borders of the atrophic regions and spared retinal islands. This allowed us to delineate the affected areas and ensure consistent identification of atrophic zones (Fig 2). Subsequently, we calculated the corresponding areas of atrophy in pixels and converted the results to square millimeters (mm^2^) using the Heidelberg images measured conversion scale (1 pixel is 291/28 microns in FAF 30°, and 1 pixel is 200/11 microns in FAF 55°). To ensure consistency, both FAF and UWF images were standardized to the same pixel scale before conversion. Performing all measurements externally in ImageJ after landmark-based distortion correction ensures that all measurements in this study are compared on a properly corrected and consistent basis across platforms.

### Measurement Analysis

We analyzed our measurement results twofold, that is, on the raw data (mm^2^), to allow direct interpretation of lesion size and focus on interpretability, and on the square-root transformed data (mm), because square-root transformed data may reduce the proportional measurement error associated with larger atrophic areas.^22^^,^^23^ Medians with interquartile ranges were presented, because they provide a more robust measure of central tendency, unaffected by outliers or skewed data.

Interrater reliability between the 2 independent graders was assessed by calculating intraclass correlation coefficients (ICCs). Intraclass correlation coefficients between graders were calculated for all measurement types, including atrophy, preserved RPE islands, and the total cohort, for each imaging modality and field of view (FAF 30°, FAF 55°, UWF-30, and UWF-55). Agreement was classified “excellent” when ICC values exceeded 0.75, and “good” when ICC values exceeded 0.60.^24^ In case of “excellent” or “good” agreement, the measurements of both graders were averaged to obtain a single mean value for each image. Additionally, Bland–Altman plots were generated to assess intermodality reliability. Because of the small sample size and the consequently limited precision of the upper and lower limits of agreement (LoAs) in these Bland–Altman plots, we also reported the 95% prediction bands around the LoAs.^25^

From the combined grader values, we tested for significant discrepancies in area measurements between FAF 30° and UWF images and between FAF 55° and UWF images, if any. Because several patients had multiple visits and both eyes were imaged, linear mixed-effects models were used to account for the hierarchical data structure. The models included random intercepts for patient ID, eye within patient, and visit within eye to account for correlations between eyes of the same patient and repeated visits. Right and left eyes were reported separately to allow for a transparent presentation of eye-level data or potential intereye variability, without suggesting a systematic difference between eyes.

Analyses were performed for the complete FAF 30° and FAF 55° data sets, as well as separately for atrophic areas and preserved RPE islands, to enable meaningful comparisons within groups with the same disease progression and to identify patterns that could be masked when analyzing the cohort. For each predefined comparison, we calculated the estimated difference, standard error, 95% confidence interval (CI), and *P* value. All hypothesis tests were 2-sided, with a significance level set at α = 0.05.

## Results

### Patient Inclusion

A total of 31 patients were identified based on our clinical database. Five patients were excluded because of a lack of genetic validation of CHM, and another 5 patients were excluded because 30° or 55° FAF images were not taken on the same day as the UWF imaging. Hence, a total of 21 patients were eligible for initial study inclusion. During image analysis, another 6 patients were excluded because of the absence of ≥5 suitable vessel alignment points required for reliable image warping. Eventually, 96 FAF images from 15 patients were included in the present study. The flowchart of patient inclusion can be seen in Figure S2 (available at www.ophthalmologyscience.org). All images analyzed in this study were captured between November 2019 and April 2025.

### Demographics

The median age of onset of symptoms in our total study population was 22 (range, 4–50) years. The median age of onset was 15 (range, 4–40) and 45 (range, 39–50) years in male and female patients, respectively. The median Snellen visual acuity measured using ETDRS-charts in this cohort, at the time of the visit, was 20/25 (range, 20/630–20/16): 20/20 in the right eye (range, 20/63–20/16) and 20/25 in the left eye (range, 20/630–20/16).

Table 1 summarizes the demographic characteristics and genetic findings of our study population.

**Table 1:** Demographics of Patients with Choroideremia Included in the Present Study

ID	Gender	Age at Baseline Imaging	Age of Onset	Age at Diagnosis	Mutation	Affected Protein	Visual Acuity	
							OD	OS
1	Male	81	40	74∗	c.517G>T	p.Glu173∗	20/63	20/200
2†	Male	20	9	9	c.1653_1658del	p.Phe552_Asn553del	20/32	20/50
3†	Male	20	9	9	c.1653_1658del	p.Phe552_Asn553del	20/32	20/32
4	Male	43	30	32	c.50-?_116+?del	p.?	20/50	20/25
5	Male	16	11	7	Deletion of the 5' end of the CHM gene	p.?	20/20	20/40
6	Male	31	10	25	c.1221_1224dup	p.Val409fs	20/16	20/20
7†	Male	21	19	19	c.[1166+1_1167-1]_[1770+1_1771-1]	p.?	20/16	20/16
8†	Male	24	22	22	c.[1166+1_1167-1]_[1770+1_1771-1]	p.?	20/16	20/20
9	Male	29	24	24	c.698C>A	p.Ser233∗	20/20	20/25
10	Female	48	39	25	c.[?_-535]_[116_?]del	p.?	20/16	20/630
11	Male	59	34	34	c.808C>T	p.Arg270∗	20/25	20/32
12	Male	35	4	4	c.1349_1349+2dup	p.?	20/20	20/32
13	Female	59	50	59	c.186C>G	p.Tyr62∗	20/20	20/16
14	Female	59	45	51	c.117-?_c.1962+?del	p.?	20/20	20/40
15	Male	26	10	12	deletion of exon 12 in the mRNA	p.?	20/32	20/40

ID = identification number; OD = right eye; OS = left eye.

∗ A delay in diagnosis was found because of misdiagnosis in this patient.

† These patients are siblings.

### Quantitative Image Grading

A total of 96 FAF images from 15 patients were graded, comprising 35 FAF 30° images, 20 FAF 55° images, and 41 UWF images.

Intraclass correlation coefficients between graders, including atrophy, preserved RPE islands, and total cohort (all measurements together), for each imaging modality and field of view (FAF 30°, FAF 55°, UWF-30, and UWF-55), ranged from 0.625 to 0.998, indicating good-to-excellent agreement for all measurement types (details depicted in Table S1, available at www.ophthalmologyscience.org).

The median area (in mm^2^) of atrophy with interquartile range was determined for UWF-30 compared with FAF 30° and UWF-55 compared with FAF 55° and is shown in Table 2. Patients presented with spared islands of atrophy or with atrophic areas. As described in the “Materials and Methods” section, we divided patients into 2 study subgroups: one including cases with atrophic areas and the other comprising eyes with spared islands (Table 3) to enable a meaningful comparison.

**Table 2:** Measurements of Areas in FAF 30° vs. UWF-30 and FAF 55° vs. UWF-55 of the Total Cohort, Median, and Interquartile Ranges

Area	FAF		UWF	
	30°	55°	30°	55°
Area in mm^2^	9.2 (3.9–14.4)	13.4 (6.2–57.1)	9.3 (4.2–15.0)	13.4 (6.5–58.5)
Area in mm^2^, right eyes	8.7 (3.2–13.1)	25.0 (5.8–55.3)	8.0 (3.3–13.8)	24.6 (5.7–56.5)
Area in mm^2^, left eyes	10.0 (4.0–20.6)	9.2 (5.6–63.6)	9.9 (4.2–20.3)	10.0 (6.2–65.2)

FAF = fundus autofluorescence; UWF = ultra-widefield.

**Table 3:** Measurements of Atrophic Areas and Spared Macular Islands of Retinal Pigment Epithelium in FAF 30° vs. UWF-30 and FAF 55° vs. UWF-55, Median, and Interquartile Ranges

Area	FAF Atrophic Areas		UWF Atrophic Areas		FAF-Spared Islands of RPE		UWF-Spared Islands of RPE	
	30°	55°	30°	55°	30°	55°	30°	55°
Area in mm^2^	30.9 (15.3–39.5)	51.4 (31.8–75.7)	29.6 (14.5–39.3)	51.8 (31.2–77.2)	7.2 (2.5–10.9)	6.3 (3.4–8.1)	7.4 (2.7–11.2)	6.6 (3.4–9.2)
Area in mm^2^, right eyes	25.9 (12.1–30.7)	39.2 (30.2–101.8)	25.7 (10.9–29.6)	38.1 (29.7–112.3)	7.2 (1.9–10.8)	5.2 (2.0–12.0)	7.3 (2.2–10.8)	5.2 (2.1–12.0)
Area in mm^2^, left eyes	37.3 (19.1–42.0)	64.3 (30.0–66.7)	37.1 (18.8–42.0)	65.4 (30.4–66.0)	9.2 (2.8–10.9)	6.3 (3.5–8.1)	9.3 (3.1–11.3)	7.4 (3.5–9.2)

FAF = fundus autofluorescence; RPE = retinal pigment epithelium; UWF = ultra-widefield.

### Atrophic Areas

When comparing FAF 30° to UWF-30, data on the atrophic areas in right eyes showed a median of 25.9 and 25.7 mm^2^, respectively. In left eyes, median areas in FAF 30° and UWF-30 were 37.1 and 37.3 mm^2^, respectively (Table 3). When comparing the FAF 55° to UWF-55, data on atrophic areas in right eyes showed a median of 39.2 and 38.1 mm^2^, respectively; in left eyes, a median of 64.3 mm^2^ in FAF 55° and 65.4 mm^2^ in UWF-55 was found. For details, see Table 3.

### Spared Macular Islands of RPE

For spared islands in right eyes, we found a median of 7.2 mm^2^ in FAF 30° and 7.3 mm^2^ in UWF-30; in left eyes, 9.2 mm^2^ in FAF 30° and 9.3 mm^2^ in the UWF-30 images were measured (Table 3). For FAF 55°, a median of 5.2 mm^2^ was found in both FAF 55° and UWF-55 for right eyes; in left eyes, we measured 6.3 mm^2^ in FAF 55° vs. 7.4 mm^2^ in UWF-55 (Table 3).

Using linear mixed-effects models in square-root transformed data, the comparison between UWF and FAF in the 30° field showed no significant difference for total cohort (estimate = 0.01; 95% CI, –0.02 to 0.04; *P* = 0.438) or for atrophy (estimate = –0.06; 95% CI, –0.14 to 0.01; *P* = 0.089), whereas preserved RPE islands differed significantly (estimate = 0.04; 95% CI, 0.01–0.06; *P* = 0.005). In the 55° field, none of the differences reached statistical significance: total cohort (estimate = 0.08; 95% CI, –0.01 to 0.18; *P* = 0.077), atrophy (estimate = 0.10; 95% CI, –0.09 to 0.28; *P* = 0.260), and islands (estimate = 0.07; 95% CI, –0.001 to 0.15; *P* = 0.054). Analyses based on the raw data resulted in highly comparable outcomes. For details, see Table S2 (available at www.ophthalmologyscience.org).

Concerning atrophic area measurements, analysis by Bland–Altman plots showed a mean bias and 95% LoA in the 30° field of –0.06 (95% LoA, –0.26 to 0.14) and in the 55° field of 0.10 (95% LoA, –0.45 to 0.65); in preserved RPE islands in the 30° field, the mean bias was 0.04 (95% LoA, –0.08 to 0.16) and in the 55° field, 0.07 (95% LoA, –0.14 to 0.28) (Fig S3, available at www.ophthalmologyscience.org). In addition, the outermost limits are presented as 95% prediction bands (Fig S3).

## Discussion

In the present study, quantitative comparison of UWF-FAF to 30° and 55° FAF imaging in CHM, after landmark-based alignment to ensure anatomic correspondence, did not reveal any significant differences in total atrophy area. Analysis of spared RPE islands, on the contrary, revealed modality-dependent differences. According to the analysis of spared RPE islands within the 30° field, UWF imaging yielded statistically significantly higher values compared with standard FAF. A similar trend was observed in the 55° field, where UWF also tended to measure larger preserved islands, although this did not reach statistical significance in our cohort. The Bland–Altman analysis showed a small mean bias, but the LoAs crossed zero, indicating variability in individual-level differences. The difference in area measurements in atrophic areas and preserved RPE islands may be explained by wavelength-dependent MP absorption. Because this effect is principally dependent on the presence of a relatively healthy central retina, preserved islands could appear smaller in blue laser light, which is largely absorbed by MP in contrast to peripheral and atrophic areas. Adding to this, some fluorophores may be better detected with green-light than with blue-light excitation, which could make spared RPE islands better detectable with UWF imaging compared with conventional FAF. Therefore, the larger spared islands observed may not necessarily indicate a truly larger area of preserved RPE but may reflect improved detection of atrophy delineation.

The comparison between UWF and conventional FAF has been addressed in several studies. Chen et al^26^ reported similar findings in Stargardt disease, where the preserved foveal island was more clearly visualized on UWF compared with conventional FAF, similarly attributed to reduced MP absorption in green-light excitation. In age-related macular degeneration, Abbasgholizadeh et al^27^ found excellent intergrader agreement (ICC ≥ 0.99) between conventional blue-light FAF and UWF green-light autofluorescence; however, they also detected small but significant absolute differences in geographic atrophy measurements, which they thought were due to differences in resolution, magnification, and light excitation. Correspondingly, Froines et al^20^ showed that baseline geographic atrophy areas were significantly larger on conventional FAF compared with UWF at baseline, although progression rates over 5 years were comparable. They proposed similar explanations, including wavelength-related effects, image distortion, and calibration differences.

When interpreting our findings in the light of previous literature, some discrepancies emerge. We found that total atrophy measurements in CHM were highly comparable across UWF and conventional FAF, despite our relatively small cohort, which is a study limitation. This contrasts with earlier studies reporting systematic differences between the modalities, even though the same equipment was used. Those studies, however, did not use the method of aligning pixel sizes before comparison, which could explain the discrepancies found. In addition, they were generally performed in geographic atrophy in age-related macular degeneration, where atrophy predominantly affects the macula. Atrophic areas in CHM are often located more peripherally, particularly in earlier stages of the disease. Another explanation for the discrepancies reported is the nature of atrophy itself. In age-related macular degeneration, the degeneration primarily involves the RPE and photoreceptors, with choriocapillaris loss often in later stages, whereas CHM is characterized primarily by loss of RPE and choriocapillaris, followed by photoreceptor degeneration, and tends to result in more extensive atrophy overall.^28^^,^^29^ In contrast, our findings in spared islands of RPE are consistent with the literature, where preserved RPE islands were measured larger on UWF compared with conventional FAF.^26^ Taken together, these findings suggest that intermodality differences can be partly explained by the greater impact of wavelength-dependent effects in the central retina. Although both techniques generally capture atrophy progression in a comparable way, baseline measurements and specific parameters in the central retina may differ between the 2 modalities. Therefore, the 2 modalities should not be considered interchangeable in clinical care or in future trials.

In light of the development of promising gene therapies and the search for suitable endpoints, particularly because several trials have failed to meet the primary endpoint of BCVA, atrophy progression should be considered a potential structural endpoint because it offers an objective and quantifiable measure of disease progression.^13^ Quantifying total retinal atrophy could be particularly useful for evaluating gene therapies that target the entire retina, such as intravitreal delivery approaches.^8^ However, this structural biomarker should not be considered a substitute for a functional endpoint but rather as a complementary outcome measure. Ultra-widefield imaging may also offer advantages in routine clinical care because it enables more extensive tracking of disease progression, thereby allowing ophthalmologists to have a more comprehensive evaluation of retinal changes. It is furthermore easier to perform, does not require pupil dilation, and is less blinding for patients, compared with conventional FAF.^9^^,^^30^^,^^31^ However, clinicians and trial designers should be aware of the potential differences in atrophy and preserved-retina measurements when interpreting results.

In conclusion, the present study showed that UWF-FAF yields comprehensive atrophy measurements in CHM, with quantitative results comparable with conventional central field FAF. This highlights the potential role of UWF imaging as a comprehensive structural biomarker for disease progression and a clinical structural endpoint in CHM trials. However, because of the differences, particularly in area measures of preserved retina, UWF and FAF modalities should not be used interchangeably.

## Ethics Statement

The requirement for study-specific informed consent was waived by the CMO Radboudumc because the study was considered minimal risk and pseudonymized data were used. However, documented consent in the electronic health record indicated that participants authorized the further use of biologic material, medical data, and imaging materials for research. Accordingly, data collection was limited to the minimum necessary information.
