# Supplementary material for: Validation of Ultra-Widefield Autofluorescence Imaging as an Endpoint for Disease Progression in Choroideremia: A Comparative Study
Source: Ophthalmol Sci. 2026 Jul 24;6(10):101331. doi: 10.1016/j.xops.2026.101331 (PMC13527496; doi:10.1016/j.xops.2026.101331)
Supplement: Supplementary file 2 [file mmc2.pdf]

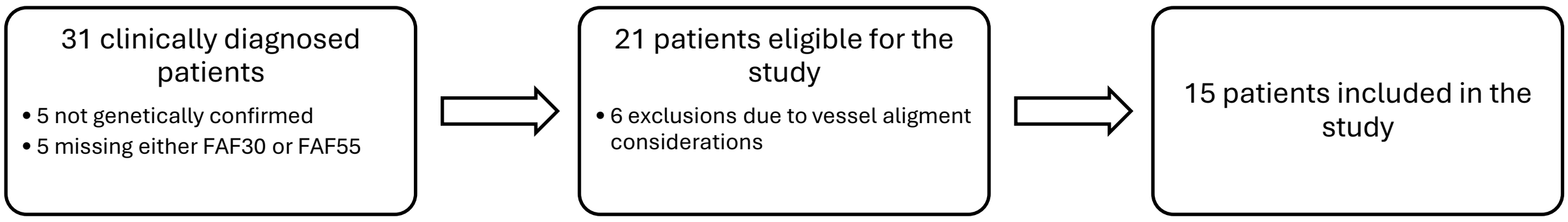

Figure S2. Flowchart patient inclusion
