# Supplementary material for: Validation of Ultra-Widefield Autofluorescence Imaging as an Endpoint for Disease Progression in Choroideremia: A Comparative Study
Source: Ophthalmol Sci. 2026 Jul 24;6(10):101331. doi: 10.1016/j.xops.2026.101331 (PMC13527496; doi:10.1016/j.xops.2026.101331)

Figure S3. Bland-Altman plots comparing UWF and conventional FAF across all outcome measures and fields. Differences are calculated as UWF - FAF; the solid line represents the bias (mean difference), and the dashed lines indicate the 95% limits of agreement (±1.96 SD). The 95% prediction bands around the upper and lower limits of agreement were as follows: for atrophy at 30°, -0.40 to 0.28; for spared islands of RPE at 30°, -0.12 to 0.20; for atrophy at 55°, -0.83 to 1.03; and for spared islands of RPE at 55°, -0.28 to 0.42.

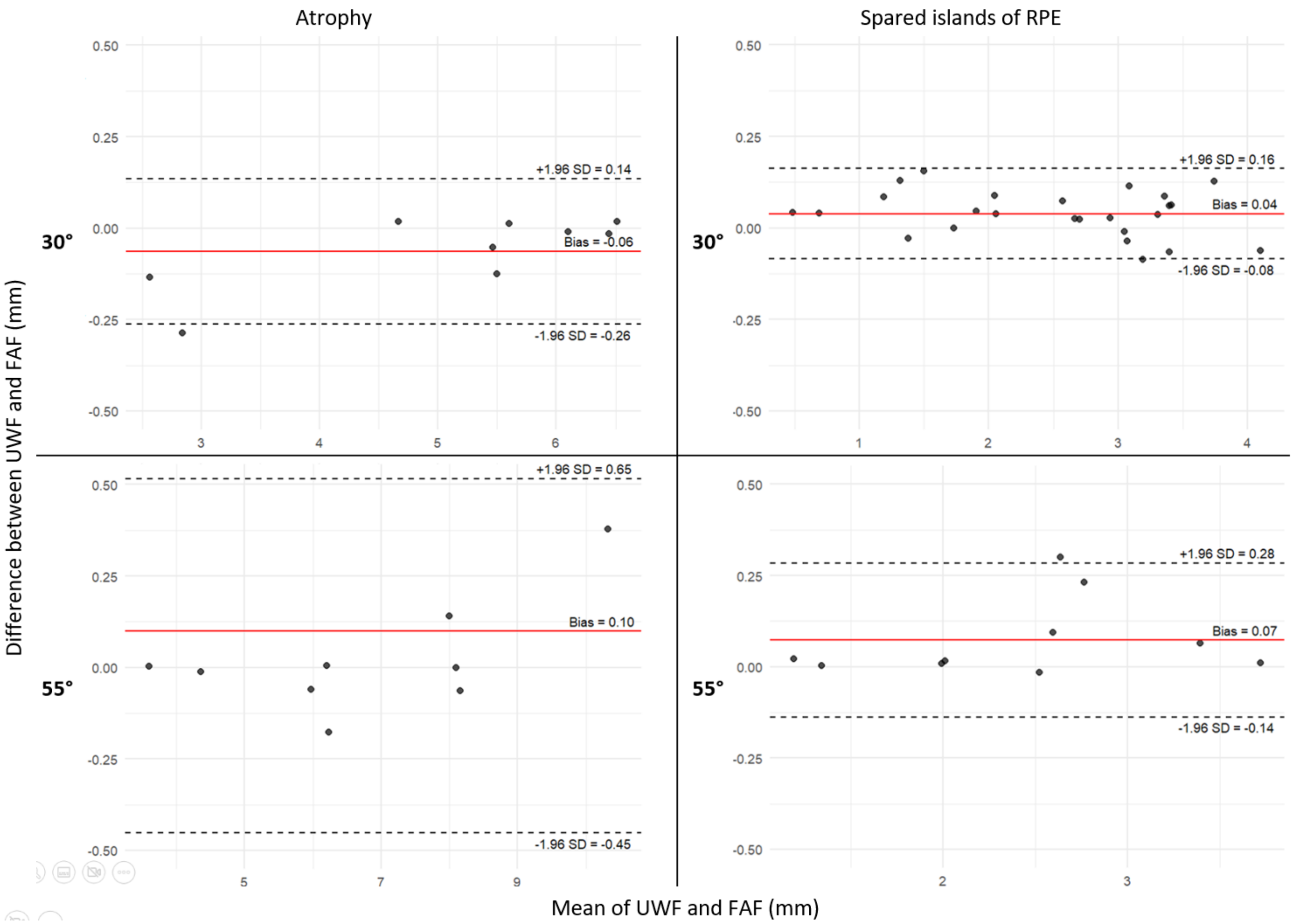

Supplement: Supplementary file 3 [file mmc3.pdf]
