# Supplementary material for: Validation of Ultra-Widefield Autofluorescence Imaging as an Endpoint for Disease Progression in Choroideremia: A Comparative Study
Source: Ophthalmol Sci. 2026 Jul 24;6(10):101331. doi: 10.1016/j.xops.2026.101331 (PMC13527496; doi:10.1016/j.xops.2026.101331)
Supplement: Supplementary file 4 [file mmc4.pdf]

Table S1. Intraclass correlation coefficients (ICCs) between graders for all measurement types (atrophy, preserved islands of RPE and total area) across imaging modalities and fields of view (FAF 30°, FAF 55°, UWF-30 and UWF-55). ICCs are shown calculated for raw measurements in mm^2 and after square root transformation.

Measured area	Imaging modality	Field of view	ICC	ICC after square root transformation
**Atrophy**	FAF	30	0.702	0.672
		55	0.992	0.995
	UWF	30	0.658	0.625
		55	0.989	0.993
**Isle**	FAF	30	0.992	0.996
		55	0.987	0.988
	UWF	30	0.98	0.989
		55	0.959	0.967
**Total**	FAF	30	0.895	0.918
		55	0.996	0.998
	UWF	30	0.866	0.887
		55	0.994	0.996
