# Supplementary material for: Validation of Ultra-Widefield Autofluorescence Imaging as an Endpoint for Disease Progression in Choroideremia: A Comparative Study
Source: Ophthalmol Sci. 2026 Jul 24;6(10):101331. doi: 10.1016/j.xops.2026.101331 (PMC13527496; doi:10.1016/j.xops.2026.101331)
Supplement: Supplementary file 5 [file mmc5.pdf]

Table S2. Measurements of areas in FAF 30° versus UWF and FAF 55° versus UWF in squared root mm2

Table 2A. Measurements of areas in FAF 30° versus UWF and FAF 55° versus UWF of the total cohort, median and interquartile ranges. UWF=Ultra-widefield.

	FAF		UWF	
	30°	55°	30°	55°
Area in squared-root mm^2	3.0 (2.0 – 3.8)	3.7 (2.5 – 7.5)	3.0 (2.0 – 3.9)	3.7 (2.5 – 7.6)
Area in squared-root mm^2, right eyes	3.0 (1.8 – 3.6)	4.9 (2.4 – 7.3)	2.8 (1.8 – 3.7)	4.8 (2.4 – 7.3)
Area in squared-root mm^2, left eyes	3.2 (2.0 – 4.5)	3.0 (2.4 – 8.0)	3.1 (2.0 – 4.5)	3.2 (2.5 – 8.1)

Table 2B. Measurements of atrophic areas in FAF 30° versus UWF and FAF 55° versus UWF, median and interquartile ranges. UWF=Ultra-widefield.

	FAF		UWF	
	30°	55°	30°	55°
Area in squared-root mm^2	5.6 (3.8 – 6.3)	7.1 (5.6 – 8.7)	5.4 (3.7 – 6.3)	7.1 (5.5 – 8.7)
Area in squared-root mm^2, right eyes	5.1 (3.4 – 5.5)	6.3 (5.4 – 10.1)	5.1 (3.2 – 5.4)	6.2 (5.4 – 10.6)
Area in squared-root mm^2, left eyes	6.1 (4.1 – 6.5)	8.0 (5.3 – 8.2)	6.1 (4.1 – 6.5)	8.1 (5.3 – 8.1)

Table 2C. Measurements of spared islands of retinal pigment epithelium in FAF 30° versus UWF and FAF 55° versus UWF, median and interquartile ranges. UWF=Ultra-widefield.

	FAF		UWF	
	30°	55°	30°	55°
Area in squared-root mm^2	2.7 (1.6 – 3.3)	2.5 (1.8 – 2.8)	2.7 (1.7 – 3.3)	2.6 (1.8 – 3.0)
Area in squared-root mm^2, right eyes	2.7 (1.4 – 3.3)	2.3 (1.4 – 3.4)	2.7 (1.5 – 3.3)	2.3 (1.4 – 3.4)
Area in squared-root mm^2, left eyes	3.0 (1.6 – 3.3)	2.5 (1.8 – 2.8)	3.1 (1.8 – 3.4)	2.7 (1.8 – 3.0)
